# Proceedings of National Medical Conference organized by Azra Naheed Medical College in collaboration with Pakistan Aspirin Foundation (April 28^th^ 2018)

**DOI:** 10.12669/pjms.344.15905

**Published:** 2018

**Authors:** Shaukat Ali Jawaid

LAHORE: Azra Naheed Medical College affiliated with Superior University Lahore organized its National Medical Conference on April 28^th^ 2018 in collaboration with Pakistan Aspirin Foundation. Harnessing the Cardiovascular Threat was the theme of the conference. Prof. Nizamuddin Chairman of Punjab Higher Education Commission was the chief guest at the inaugural session. Speaking at the occasion, he stated that we need to create medical institutions which promote research culture besides improving the quality of research. Doctors should improve documentation; maintain record of their patients which is extremely helpful in conducting research. We also need to promote preventive measures, use of low dose Aspirin for longer periods and change our lifestyle besides using healthy diet which will overcome numerous diseases.

**Figure:**
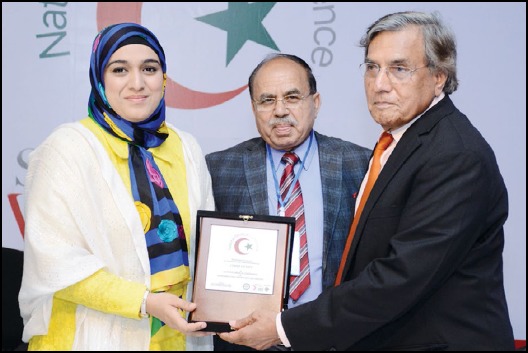
Prof. Sumaira Rehman Rector of Superior University alongwith Prof. M. Akbar Chaudhry Principal Azra Naheed Medical College presenting a memento to Prof. Nizamuddin Chairman PHEC at the inaugural session of National Medical Conference held on April 28th 2018.

Continuing Prof. Nizamuddin said that we need to modify the known risk factors for cardiac diseases, control hypertension, diabetes mellitus, high cholesterol, obesity, physical inactivity, smoking, stress and unhealthy diet. Those with major risk factors are likely to suffer from various heart diseases. National Health Survey conducted in 2011 had shown that over 18% of our population above the age of eighteen years suffer from hypertension, Pakistan at present is at No. six as regards the prevalence of diabetes which is increasing and by 2020 we may be the No.4 country in the world with highest number of people with diabetes. At present about 25% of people over the age of forty five years suffer from diabetes mellitus and prevalence of smoking is reported to be between 14-21% in adolescents and adults. Healthy diet, active life style, staying positive all-day can go a long way in reducing the risk factors for cardiac diseases and diet also helps in attaining a healthy lifestyle. Obesity is caused when we consume more calories than our body burns and abdominal obesity is a major risk factor. He laid emphasis on the fact that exercise alone will not control or reduce weight and modifying the diet was also important.

Medical institutions should promote research culture & improve quality of research-Prof. Nizamuddin

Speaking about the higher medical education in Pakistan, Prof. Nizamuddin said that it suffers from some structural problems. We need to establish training institutions for teachers. So far what has been going on is that soon after selection, people start teaching, they need to be trained. We in the Punjab Higher Education Commissions have made a policy that teaching and training should go hand in hand. It is of no use to start teaching without any training. To enhance the quality of higher education, PHEC has plans for post doctorate PhD Fellowship. It is a split programme wherein one can work for two years in Pakistan and then work for one year overseas at leading institutions working under supervisor. We in PHEC are concerned about the quality of education as quality has been compromised which was reflected during the defense of the PhD thesis by the candidates.

Continuing Prof. Nizamuddin said that while we have established numerous medical and dental colleges producing doctors and dentists but training of support staff like nurses, technicians, dental hygienists has been neglected. Ideally there should be five support staff per doctor and for every dental surgeon there should be two to three dental hygienists and no one should be given license to practice without these essential requirements. Doctors should not do everything themselves. They should also involve the senior medical and dental students in research. Fresh medical graduates should also be trained if they wish to establish their own practice. He commended the pre-conference workshop idea which must have enabled to train many participants, he remarked.

**Figure:**
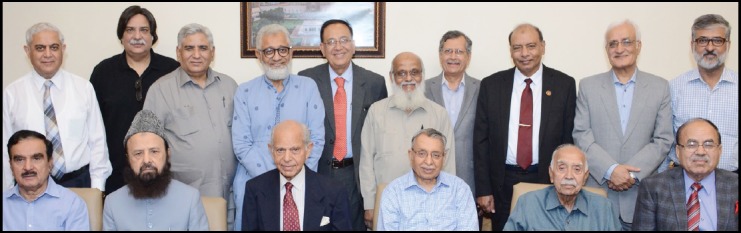
Pakistan Aspirin Foundation had a meeting at Lahore on April 27th, 2018. Group photograph taken during the council meeting shows sitting from (L to R) Maj. Gen. Ashur Khan, Prof. Abdus Samad, Dr. Shaukat Malik, Prof. Ejaz Ahmed Vohra, Prof. Mahmood Ali Malik, Prof. M. Akbar Chaudhry. Standing from (L to R) Prof. Saulat Siddique, Dr. Dawar Majeed, Mr. Shaukat Ali Jawaid, Prof. Javed Akram, Prof. M. Ishaq, Prof. A. Rasheed, Prof. Mansoor Ahmed, Prof. Naeem Aslam, Prof. Shahbaz Kureshi and Mr. Kashif Riaz from Acto Laboratories which sponsor CME Activates of the Foundation.

Referring to the use of Aspirin to prevent heart diseases, he pointed out that use of low dose Aspirin for longer period reduces the morbidity and mortality from many disease including heart diseases. Our hospitals are gold mine of data which can be used for research. We also need to study whether genetic diseases can be prevented. Involving the community in preventive measures will be quite helpful. Countries like Cuba and Sri Lanka have improved their health indicators tremendously just by concentrating on primary healthcare. Serious complications can also be prevented if the diseases are tackled in the initial stages. We also need to study the relationship between different chronic diseases, Prof. Nizamuddin remarked.

Preventive measures, use of low dose Aspirin & change of life style can overcome many diseases

Earlier **Dr. Muqqadas Rehman** Director Azra Naheed Centre for Research & Development said that we feels honoured to be part of this conference. Cardiovascular diseases is a threat worldwide and it is also a great concern in Pakistan. According to reports almost 40% of death in Pakistan is related to heart diseases, hence we need to find indigenous cost effective solutions to these problems. Heart is an integral part of our body and we need to ensure its efficient functioning. Speaking on a lighter note, she remarked that “Heart is on the left side but it cannot be left ignored”. Azra Naheed Centre for Research and Development is playing its role in ensuring healthier society through advanced research.

**Prof. Sumaira Rehman** Rector of Superior University/Azra Naheed Medical College in her address pointed out that the basic objective of this conference was to create awareness about the diseases most of which are preventable. We need to join hands to control hypertension and diabetes which increase the burden of heart diseases. We must come up with some innovative solutions which are essential for scientific progress in this particular field. Superior University, she further stated has started Health Care Entrepreneurship Programme for its medical students to enable and empower them to deal with these challenges. Through such programmes we can inculcate the entrepreneurial mindset in our youth so that they can come up with creative and innovative measures in this field. Provision of Basic Healthcare in Pakistan still remains a question mark and we need to make a real contribution to basic healthcare in Pakistan. She also appreciated the role of Azra Naheed Centre for Research and Development for promoting research culture at the institution. **Prof. Mohammad Akbar Chaudhry** Principal of AZNMC in his welcome address thanked the chief guest and the distinguished participants for gracing the occasion with their presence.

Management plan for CVD should include Aspirin daily, BP control, Cigarette smoking cessation, management of diabetes & pre-diabetes, regular exercise and healthy diet - Prof. Ishaq

**Figure:**
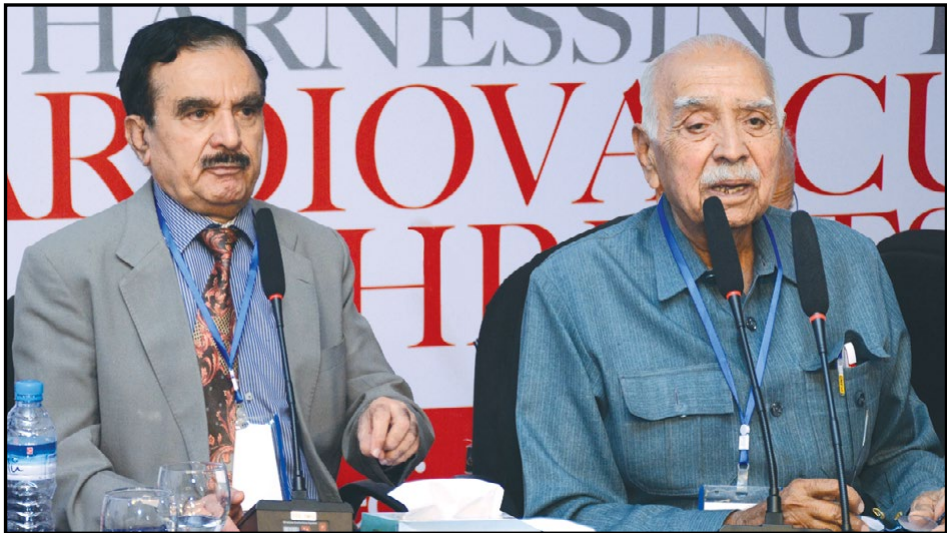
Prof. Mahmood Ali Malik along with Maj. Gen.Ashur Khan chairing the first scientific session during the National Medical Conference held at AZNMC in collaboration with Pakistan Aspirin Foundation.

**First Scientific Session**

Prof. Mahmood Ali Malik along with Maj.Gen. Ashur Khan chaired the first scientific session which was moderated by Prof. Zahid Latif. **Prof. M. Ishaq** from Karachi was the first speaker who talked about epidemic of cardiovascular diseases. Cardiovascular diseases, he said claim more than eighteen million deaths every year which accounts for one third of total global mortality. The developing world has not yet controlled communicable diseases but it is also faced with non-communicable diseases epidemic. South Asia is the worst affected. Within South Asia, low income and middle class is the worst affected. Coronary heart disease accounts for 47% mortality in men and 38% mortality in women whereas stroke accounts for 34% and 37% of mortality in men and women respectively.

In South Asia there is high prevalence of diabetes mellitus, abnormal LDL-HDL ratio, and early onset of the disease. Moreover there is high prevalence of obesity, hypertension, and tobacco use besides physical inactivity. South Asian also has the emerging risk factors like insulin homocysteinemia and raised CRP besides fibrinogen levels. Solution to all this lies in primary prevention. He advocated creating awareness and life style changes in the community to control this epidemic. In Pakistan hypertension, diabetes, obesity and tobacco consumption is rising. Migration from rural to urban areas has made the situation worse. Other challenges include rising poverty, illiteracy and meager resources. He suggested an integrated approach by healthcare professionals, public and the policy makers to achieve the desired results. The strategic plan could include Aspirin daily, Blood Pressure control, Cigarette smoking cessation, management of diabetes and pre-diabetes, regular exercise and healthy diet, he remarked.

**Prof. Muhammad Atif Qureshi’s** presentation was on Diabetes Mellitus & Hypertension: Two Bad Companions. Many patients, he said, come to know that they suffer from diabetes or hypertension when they develop life threatening complications. Diabetes and hypertension both are having rapid increase in low and middle income countries. It is essential that we diagnose diabetes and hypertension as early as possible and initiate treatment. It will help avoid serious complications. Weight reduction and life style modification were also important to manage hypertension. Studies have shown the prevalence of diabetes to be 28.3% in urban and 25.3% in rural areas. The prevalence of pre-diabetes was 15.5% in urban and 13.9% in rural areas. Similarly the prevalence of hypertension was 24.7% who are newly diagnosed while known hypertensives account for 27.9%. The overall prevalence of hypertension is reported to be 52.6%. Obesity which is another important risk factor is also increasing while the overall prevalence of dyslipidemias was 93.2%.

Hypertension and diabetes are two leading risk factors for atherosclerosis, its complications include heart attack & strokes-Prof. Atif Qureshi

Speaking about the important complications of diabetes Prof. Atif Qureshi mentioned dental diseases, complications of pregnancy, hypertension, diabetic retinopathy, amputation, nervous system diseases besides heart diseases and stroke. Hypertension affects about 70% patients with diabetes, hypertension is twice common in persons with diabetes, three quarter of people suffering from diabetes also suffer from hypertension while one in three persons suffering from Type-I diabetes is also suffering from high blood pressure. Hypertension and diabetes are two leading risk factors for atherosclerosis and its complications includes heart attack and strokes. In case the person is suffering from both these diseases, the effect of one disease tends to make the other disease worse. Diabetes and hypertension co-exist as they share similar risk factors like obesity, unhealthy diet and sedentary life style. They both affect blood vessels, heart, brain, eyes and kidneys.

Continuing Prof. Atif Qureshi said that certain sub-groups have serious risks like pregnant women are at risk of pre-eclampsia, children suffering from Type-1 diabetes and hypertension may develop end organ disease. Increased incidence of Type-2 diabetes in children is serious because it may lead to cardiovascular risk factors in early life which will accelerate atherosclerosis with aging. The solution lie in diagnosing diabetes and hypertension early, aggressive treatment to prevent associated micro vascular and macro vascular morbidity and mortality. Each 10mmHg decrease in mean systolic blood pressure will lead to 12% reduction in the risk of complications, 15% reduction in death related to diabetes, 11% reduction in myocardial infarction and 13% reduction in micro vascular complications. While drug therapy is required to manage diabetes and hypertension, lifestyle modification, weight management, are the most important components to reduce hyperglycemia and control of blood pressure.

Low Dose Aspirin is effective in pre-eclampsia, HELP syndrome and it is also indicated in high risk pregnancies - Prof. Javed Akram

**Prof. Javed Akram** discussed in detail as to how Aspirin works. He talked at length about platelet activation, aggregation and clot formation. The physicians should be careful about dangerous bleeding. It is usually related to dose. However, the low dose Aspirin which we recommend for long term use i.e. 75mg to 100mg is quite safe and effective. Those who are taking low dose aspirin regularly should not donate blood. Low dose Aspirin, he further stated has also proved effective in repeated miscarriages, recurrent abortions and in Antiphospholipid Syndrome. It also reduces infertility by 12-15% as reported in some studies. He suggested use of low dose Aspirin in pre-eclampsia as well as HELP syndrome. Low Dose Aspirin therapy is also indicated in high risk pregnancies. Studies have also reported 50% clinical response in Antiphospholipid syndrome. He cautioned that one should be mindful of Aspirin resistance while there are some patients who do not respond to Aspirin which can be termed as non-responders. He concluded his presentation by stating that Aspirin remains a wonder drug, we come to know about its new and emerging indications regularly and in the times to come we will come to know much more about it.

In his concluding remarks **Maj. Gen.Ashur Khan** commended the presenters and opined that most of them have stressed the importance of life style modifications. **Prof. Mahmood Ali Malik** stated that he had learnt many new things as the presentations were very informative. Aspirin is one of the most inexpensive easily available drugs which was quite useful. It is not only helpful in hypertension but it can also prove lifesaving in acute myocardial infarction. While we have not yet controlled the communicable diseases, we are also faced with the non-communicable diseases. The developed world is now looking after the NCDs. Abdominal obesity, he opined was very dangerous as it is associated with metabolic complications. Cholesterol less than two hundred will protect from cardiovascular diseases but less is better. However, if it is less than one hundred, it can lead to colon cancer. As regards insulin resistance we need to find out whether it leads to diabetes or is the insulin resistance cause of obesity, he remarked.

Abdominal obesity was very dangerous as it is associated with metabolic complications-Prof. Mahmood Ali Malik

**Second Session**

Prof. Abdus Samad along with Dr.Shaukat Malik chaired this session which was moderated by Prof Atif Qureshi. **Prof. Mansoor Ahmad** made a presentation on primary prevention of cardiovascular disease. Aspirin, he opined, does save life but it may not improve patency. He also talked about whether Aspirin was as effective in primary prevention as in secondary prevention, risk stratification and total cardiovascular risk. High risk patients, he said, respond better. Cost of managing cardiovascular diseases, Prof. Mansoor Ahmad remarked is going up and intervention procedures is going down as the disease is being controlled. Age, he opined was a major risk factor. Use of tobacco and all other smoking, high LDL, hypertension, low HDL, family history of coronary heart disease and age were the major risk factors. Obesity was another important risk factor besides physical inactivity. One has to calculate ten years risk assessment and if it is above five or higher, the patient is diabetic and above forty years of age, it is a high risk patient. Diabetics have high mortality from Acute Myocardial Infarction. Even in post MI the chances of mortality remain high. Those who have more exercise, reduce their risk. Sedentary behaviour is a risk factor.

Diabetics have high mortality from AMI, even in post MI the chances of mortality remain high- Prof. Mansoor Ahmad

Primary prevention involves the prevention of risk factors causative of the disease thereby reducing the likelihood of development of the disease. Secondary prevention refers to the prevention of or recurrence of disease in those who already have the symptoms. Though there are some reports about the beneficial effects of Aspirin in primary prevention but its role still remains controversial. In some patients intracranial GI bleed risk outweighs the benefits. USPS Task Force recommends Low Dose Aspirin for people between 50-69 years of age if they have low bleeding risk, have 10% high risk of CVD event, life expectancy is ten years or more, if they are prepared to take Aspirin for ten years or more, diabetics who are above fifty five years of age and diabetic women who are above sixty years of age.

**Figure:**
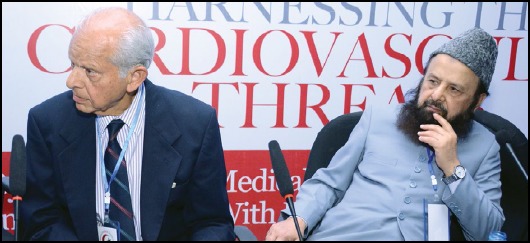
Prof. Abdus Samad along with Dr. Shaukat Malik chairing the second scientific session during the National Medical Conference held at AZNMC in collaboration with Pakistan Aspirin Foundation.

Prof. Mansoor Ahmad concluded his presentation by stating that primary prevention has proved successful in many societies as evidenced in reduction in incidence and prevalence of CVD. Over the years mortality from CVD has gone down. Successful programme needs to be coordinated between individuals, societies and the Government. Statins are very important medications in all those who have ten years risk of 7.5% or more. It is important to control high blood pressure; lipids should be brought down to acceptable levels using Statins, cessation of tobacco use, smoking, physical activity should be promoted. Primary prevention should start in childhood and it needs to be promoted in all cultures and above all obesity of any form should be discouraged.

Use of Aspirin and other Antiplatelet in secondary prevention of cardiovascular disease was highlighted by **Prof. M. Akbar Chaudhry**. CVD, he said remains the No.1 cause of death globally. In Pakistan one in four people may have underlying CAD in the urban population. Pakistani patients, he further stated, are about ten years younger than patients seen in the West. He then highlighted the high prevalence of the known risk factors like hypertension; we have more than 6.9 Million people suffering from diabetes. We have high prevalence of dyslipidemias, obesity, metabolic syndrome and smoking. A more recent survey by BIDE conducted in collaboration with PHRC, Government of Pakistan and DAP showed that newly diagnosed hypertensives were 24.7%, known hypertensives accounted for 27.9%, thus overall prevalence of hypertension in Pakistan was 52.6%. We have overall prevalence of dyslipidemias of 93.2%.

Speaking about the management of Acute Myocardial Infarction Prof. Muhammad Akbar Chaudhry suggested admission to coronary care unit, relief of pain with Morphine and Nitrates maintain IV line, monitor ECG and BP, use of thrombolysis, anticoagulants, Aspirin, beta blockers, calcium channel blockers and ACE Inhibitors. Treat complications like dysrhythmia, LVF, CCF and shock besides planning for late complications. Highlighting the role of Aspirin, he pointed out that when used in AMI within six hours it reduces mortality by 23%, use of Streptokinase within six hours will reduce mortality by 25% but if both are used within six hour, the motility reduction could be increased to 46%. This all proves that Aspirin was poor man’s streptokinase which is not only cheap but also do not require IV infusion and monitoring. Despite so many benefits, Aspirin, Prof.Akbar Chaudhry remarked remains under used in TIAs, MI, and CAD. We need to encourage the use of Aspirin in secondary prevention especially in those who are high risk patients. He also referred to the findings of Aspirin Awareness and Usage Study conducted by Pakistan Aspirin Foundation in 2005 which had showed that only 16% patients took aspirin at home when they suffered from chest pain, only 20.8% of patients were prescribed Aspirin by their family physicians and just 50% of ACS patients were prescribed Aspirin at the time of discharge. Referring to the benefits of Aspirin in secondary prevention, he mentioned that it reduces serious vascular events as under:

Despite proven benefits, Aspirin remains under used in TIAs, MI, and CAD - Prof. Akbar Chaudhry

- 46% in unstable angina
- 33% in-stable angina
- 23% in peripheral arterial disease
- 53% in patients undergoing angioplasty.

Numerous other studies have also showed reduction in non-fatal MI by 32%, Non-fatal stroke by 27%, and Total CV mortality by 15% and improvement in vascular events by 25%. As regards risk reduction for morbidity and mortality, for every one thousand patients per year, use of Aspirin will result in 30-40 patients with recent MI or stroke who will avoid a vascular event, 15-20 patients with angina will avoid an event and about 15 high risk patients will also avoid an event. In patients suffering from Ischemic stroke or TIA, the recommended dosage of Aspirin was 150-300mg per day and it must be initiated as early as possible and continued indefinitely. Newer antiplatelet should be used when Aspirin is contra-indicated. In high risk patients Dipyridamol or Ticlodipine or Clopidogrel may be added but one has to be careful about potential adverse effects and the cost. Meta-analysis of six studies which enrolled 95,456 individuals showed the use of Aspirin in stroke in women resulted in 17% reduction in stroke in women and 14% reduction of stroke in men. Aspirin 150-300mg given within 48 hours of acute ischemic stroke is the drug of choice with low risk of adverse events. Chinese Acute Stroke Trial and International Stroke Trials performed in 1997 had also showed that Aspirin reduces the risk of early death in acute stroke by 5/1000 and recurrence & death by 10/1000. The relative risk reduction of stroke and death with Aspirin was 31% while Aspirin dose of 75-325mg daily was the drug of choice in prevention of secondary stroke as reported in SALT study in Lancet in 1991 and Neurology in 1998. CURE study had also confirmed 20% relative risk reduction with the use of Aspirin in Myocardial Infarction, Stroke and cardiovascular death as compared to Clopidogrel after a twelve month follow up.

Aspirin 150-300mg given within 48 hours of acute ischemic stroke is the drug of choice with low risk of adverse events

**Prof. Ejaz Ahmad Vohra** discussed the use of Aspirin and Statins in cardiovascular diseases. He pointed out that both these drugs are used to prevent Atherosclerotic vascular disease using dual mechanism of Antiplatelet action of Aspirin and Anti-inflammatory action of Statins. Various studies have confirmed inflammatory basis of Atherosclerosis and Aspirin has probable beneficial effects on Cytokines and endothelial dysfunction. Numerous studies have confirmed beneficial effects of Statins in reducing CRP and LDL cholesterol confirming inflammatory causation of atherosclerosis.

Various trials Prof. Ejaz Ahmad Vohra further stated have demonstrated reduction in cardiovascular events in acute coronary syndrome when Statins are added within forty eight hours of the initial management. A number of studies confirm that Aspirin and Statins reduced adverse events and mortality in secondary prevention. Aspirin and Statins remain the first line therapy for acute coronary syndrome. With the use of Aspirin and Statins, diabetics without CHD have the same risk for myocardial infarction as non-diabetics. He also referred to the use of Polypill in prevention of cardiovascular disease and said that randomized trials have shown that Polypill combined with Statins, one or more antihypertensive and Aspirin improve treatment adherence rates and safely reduces cardiovascular risk factors in patients with established cardiovascular disease. However, there is another viewpoint that a healthy lifestyle can achieve the same result and Polypill was not a replacement for healthy life style. Another study has shown that in patients with acute MI complicated by heart failure, prescription of Aspirin and Statins either alone or in combination is associated with better long term survival. Speaking about adverse effects of Aspirin, Prof. Ejaz Ahmad Vohra remarked that Aspirin is known to cause hemolytic anemia in people who have the genetic disease glucose-6-phosphate dehydrogenase deficiency. Aspirin should not be taken by people who are allergic to ibuprofen or naproxen. Aspirin and Statins is a logical combination in prevention and management of atherosclerotic vascular disease. In addition there are emerging uses in Chemoprevention of Cancer, he remarked.

Aspirin and Statins remain the first line therapy for acute coronary Syndrome-Prof. Ejaz Ahmad Vohra

**Prof. Abdus Samad** gave details of various Guidelines on the use of dual antiplatelet therapy. Aspirin, he said, was a more selective inhibitor of COX-I, thereby inhibiting platelet aggregation. On the other hand, Clopidogrel and ADP receptive blocker has been shown to be more effective than Aspirin in reducing the primary end-points for secondary prevention. Combination therapy of DAPT is now used in multiple clinical conditions for prevention of platelet aggregation, achieving better clinical end-point results. The dual antiplatelet therapy includes combinations of Aspirin and Clopidogrel, Aspirin and Prasugrel, Aspirin and Ticagrelor, Aspirin and Dipyridamol, Aspirin and Cilastazol. The CURE study which enrolled 12,562 ACS patients compared Aspirin vs. Aspirin plus Clopidogrel. This, Prof. Samad said showed 2.1% absolute risk reduction. Talking about the Ischemic burden Prof. Samad mentioned age more than sixty five years, second myocardial infarction, diffuse diabetes mellitus, stent length more than 60mm, patients with three or more stents implanted, at least three lesions stented, last patent coronary artery stented, Bifurcation two stent strategy and the use of stent within the stent.

DAPT is now used in multiple clinical conditions for prevention of platelet aggregation, achieving better clinical end-point results-Prof. Abdus Samad

**Figure:**
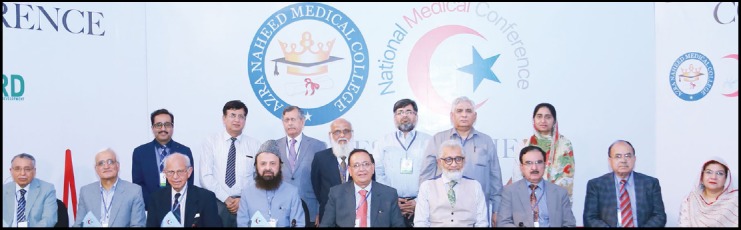
Group Photograph taken at the concluding session of the National Medical Conference of AZNMC held on April 28th from (L to R) are Prof. Ejaz Ahmad Vohra, Dr. Shahbaz Kureshi, Dr.Shaukat Malik, Prof. Abdus Samad, Prof.M.Ishaq, Prof. Javed Akram, Maj. Gen. Ashur Khan, Prof. M. Akbar Chaudhry and Prof. Naela Tarique. Standing second row from (L to R) Prof. Atif Qureshi, Prof. Saeed, Prof. Mansoor Ahmad, Dr. A. Rashid, Dr. Zahid Latif, Mr.Shaukat Ali Jawaid and Prof. Shaheen Kausar.

Continuing Prof. Abdus Samad said that in NSTE-ACS patients in whom coronary anatomy is not known, it is not recommended to administer Prasugrel. Prasugrel is also not recommended in patient’s age seventy five years or above and those with body weight of less than 60kg or history of previous intra-cranial hemorrhage, previous ischemic stroke or TIA. Ticagrelor is contra indicated in patients with previous intra-cranial hemorrhage or those with on-going bleeds. It is not recommended to discontinue DAPT within the first month of treatment in patients undergoing elective non-cardiac surgery. Routine platelet function testing to adjust DAPT before or after elective stenting is not recommended. Prasugrel is also not recommended in medically managed patients. It is recommended to continue Aspirin throughout peri-operative period and to resume DAPT as soon as possible in patients undergoing elective cardiac or non-cardiac surgery.

**Panel Discussion**

New and emerging indications for Aspirin Therapy were highlighted in the panel discussion. The members of the expert’s panel included Prof. Javed Akram, Dr.Shaukat Malik, Dr. Shahbaz Kureshi, Maj. Gen.Ashur Khan, Prof. Naela Tarique, Prof. Shaheen Kausar and Prof. M. Saeed. This was moderated by Prof. Akbar Chaudhry. During the discussion use of Aspirin in patients with bad obstetric history, recurrent miscarriages, infertility, Anti Phospholipid Syndrome, HELP Syndrome Pregnancy Induced Hypertension, colon cancer were highlighted.

**Use of Aspirin in Obstetrics & Gynaecology**

Prof. Naela Tarique participating in the discussion sated that Aspirin plays a major role in many Obstetrical and Gynecological conditions. Some important ones include Pre-Eclampsia, Intrauterine Growth Retardation, Recurrent Pregnancy Loss and Deep Vein Thrombosis.

## Pre-Eclampsia

Pre eclampsia, she said, is development of hypertension in pregnancy at or after 20 weeks. Blood pressure is equal to or greater than 140/90 plus protein urea is greater than or equal to 3g / 24 hours in urine. It effects 2-8 % of pregnancies and was the second leading cause of maternal mortality worldwide. In normal pregnancy prostacyclin increases markedly. It inhibits platelet aggregation and causes vasodilatation. In pre-eclampsia there is dominance of Thromboxane A2 over prostacyclin that is strong vasoconstrictor. According to CLASP (Collaboration Low dose Aspirin Study in Pregnancy), prophylactic use of Aspirin decreases pre-eclampsia in 12% of pregnancies. According to US preventive services task force prophylactic, 60-150mg Aspirin results in reduction in Pre-Eclampsia by 24 %. Preterm birth by 14% & intrauterine growth retardation by 20%

Aspirin reduces Pre-Eclampsia by 24%. Preterm birth by 14% & intrauterine growth retardation by 20% - Prof. Naela Tarique

## Intrauterine Growth Retardation

Referring to Intrauterine Growth Retardation Prof. Naela Tarique said that it results when estimated fetal weight is below the 10^th^ percentile for its gestational age as determined through ultrasound. Its incidence is 10% in general population. Its pathogenesis is inadequate trophoblastic invasion of spiral arteries leading to decreased perfusion of intra cotyledon space resulting in abnormal development of terminal villi. It decreases blood flow and impairs transfer of oxygen and nutrients to the fetus. Aspirin decreases occurrence of Intrauterine Growth Retardation in women at increased risks. For this condition Aspirin 75-80 mg Tablet daily is the recommended dose in patients at high risk for pre-eclampsia. ACOG (American College of Gynecology), WHO (World Health Organization), NICE guidelines (National Institute for Health and Care Excellence) and AHA (American Heart Association all recommend prophylaxis with aspirin in Intrauterine Growth Retardation, she added.

## Recurrent Pregnancy Loss

Recurrent Pregnancy Loss Prof. Naela Tariq stated means three or more consecutive miscarriages before 10 weeks of pregnancy or one or more morphologically formed fetal loss after 10 weeks of gestation or one or more preterm birth before 34 weeks due to placental pathology. There are multiple causes of recurrent pregnancy losses among which treatable cause include Antiphospholipid Antibody Syndrome. Mechanism includes activation of compliment pathway at maternal fetal inter-phase resulting in local inflammatory response and thrombosis of utero placental vasculature. Aspirin inhibits platelet cyclooxygenase irreversibly and decreased production of thromboxane A2 and causes vasodilatation thus preventing thrombosis.

## Deep Vein Thrombosis

Speaking about DVT, she said, pregnancy is a pro thrombotic state because it has all components of Virchow’s Triad that is stasis, vessel wall injury and hyper coagulability thus causing thrombosis. Deep Vein Thrombosis is a blood clot that forms in a vein deep in the body mostly in lower leg or thigh. Pathogenesis of clotting mechanism in vein is tissue factor that leads to thrombin generation and activation of white cells. This leads to platelet aggregation and fibrin formation. Platelet aggregation plays an important role in development and propagation of deep vein thrombosis. Aspirin blocks Cyclooxygenase– II and results in decreased platelet aggregation. It also causes inhibition of local expression of inflammatory mediators including cytokines and Cyclooxygenase–II and thus inhibits thrombotic process.

## Aspirin in Ophthalmology

**Prof. Dr. Muhammad Saeed** participating in the panel discussion spoke about of Aspirin in ophthalmology and pointed out that it seems we are far behind in getting benefits of this wonder drug. In mid & late 90’s few studies were conducted on the role of Aspirin for the prevention of cataract. In these studies, the postulated mechanism by which aspirin could affect progression of cataract was; acetylation of lens protein & decrease of glycaemia. None of these studies were specifically designed to test the effect of aspirin on cataract. Strangely enough to date very few if any studies have been conducted to further highlight the role of aspirin in preventing cataracts. He was of the view that there is a strong need to carry out still more studies to explore the potential benefits of this wonder drug for the prevention of one of the most common causes of reversible blindness and decrease to some extent the ever rising burden of cataract surgery all over the world particularly in developing countries. Other potential areas where benefit of Aspirin can be explored are treatment of pterygium, certain types of conjunctivitis especially allergic conjunctivitis, uveitis and control of postoperative inflammation, Prof. Saeed remarked.

Summing up the discussion Prof. Samad opined that in today’s era we need sound evidence before recommending anything. It was also suggested that we may look into undertaking some local studies regarding use of Aspirin in some of these emerging indications. The importance of having Evidence Based Guidelines was also highlighted.

